# Airway strategies for lung isolation in a patient with high-velocity nail gun injuries to the right cardiac ventricle and floor of the mouth: a case report

**DOI:** 10.1186/1752-1947-7-137

**Published:** 2013-05-28

**Authors:** Herman Lim, Laurence Weinberg, Chong Oon Tan, Stanley Tay, Constantine Kolivas, Philip Peyton

**Affiliations:** 1 Department of Anaesthesia, Austin Hospital, 145 Studley Road, Heidelberg, VIC, 3084, Australia

**Keywords:** Anaesthesia, Nail gun injury, Airway management, Cardiopulmonary bypass

## Abstract

**Introduction:**

We report a case of deliberate self-harm in which three three-inch nails were fired from a nail gun resulting in mandibular fixation and two penetrating injuries to the right cardiac ventricle. This combination of high-velocity penetrating injury has not been previously described.

**Case presentation:**

A 69-year-old Caucasian man with a medical history of chronic depression was brought to hospital after a failed suicide attempt. The attempt consisted of self-asphyxiation with car exhaust fumes and shooting himself thrice with a three-inch nail gun. He sustained a penetrating nail injury to the floor of his mouth, effectively pinning his mouth closed, and penetrating injuries to the right ventricular free wall and at the junction of the right atrioventricular septum. The patient required emergency surgery with requirements for thoracotomy and sternotomy, lung isolation and cardiopulmonary bypass.

**Conclusions:**

This is the first reported case of a combination high-velocity penetrating nail gun injury to the face and the right cardiac ventricle. This rare case offers airway strategies to accommodate the surgical requirement for lung separation for penetrating chest trauma in a patient with iatrogenically limited mouth opening.

## Introduction

A nail gun has the ability to concatenate the energy of multiple hammer strikes into a single focused shot resulting in nail velocities comparable to a small-calibre handgun. We report a case of deliberate self-harm in which three three-inch nails were fired from a nail gun resulting in mandibular fixation and two penetrating injuries to the right cardiac ventricle. This combination of high-velocity penetrating injuries has not been previously described. We report the challenging considerations required to accommodate the surgical requirement for lung separation for penetrating chest trauma in a patient with iatrogenically limited mandibular excursion.

## Case presentation

**Figure 1:**
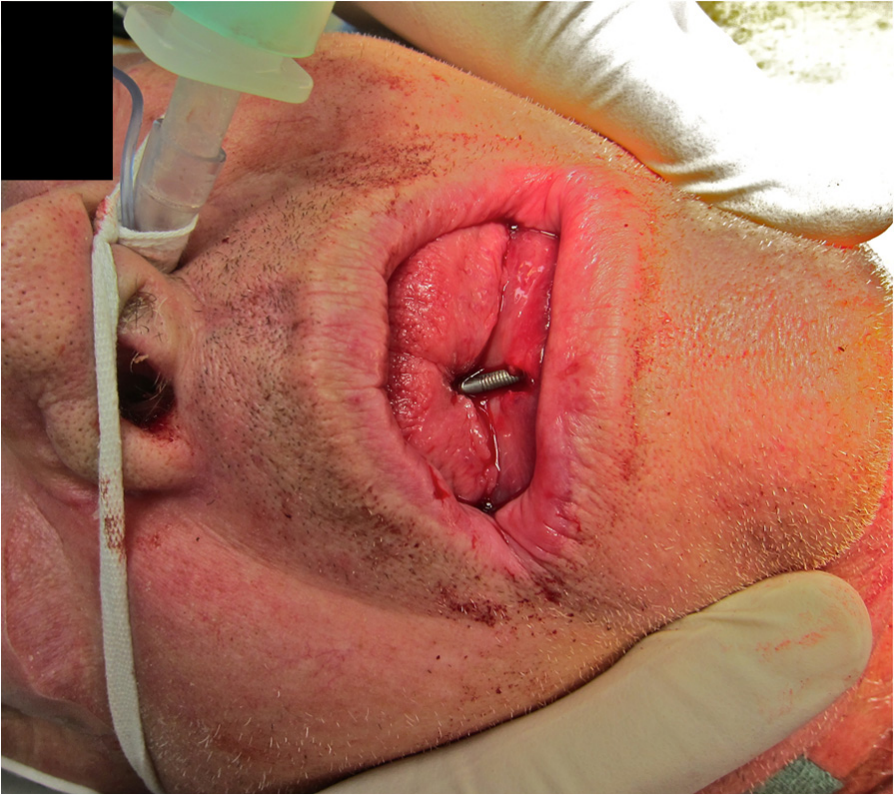
High-velocity nail gun injury that entered through the submental triangle penetrating through the floor of the mouth, tongue, dentures and hard palate, severely limiting mandibular excursion.

A 69-year-old Caucasian man, a retired builder, with a medical history of chronic depression was brought to hospital after a failed suicide attempt. The attempt consisted of self-asphyxiation with car exhaust fumes and shooting himself thrice with a three-inch nail gun. The initial shot was directed upward through the submental triangle behind the chin. It pierced his tongue, upper denture plate and hard palate, effectively pinning his mouth shut (Figure 1). The subsequent two shots were fired posteriorly via the fourth intercostal space immediately left of the sternum.

On admission to the emergency department the patient was distressed but haemodynamically stable. An arterial blood gas sample was unremarkable apart from mildly elevated carboxyhaemoglobin levels. An examination revealed nail gun entry wounds on his left anterior chest wall over the precordial area, and at the submandibular area under his chin, pinning his mouth closed. The nails within the thorax could not be confidently located on chest X-ray; however, transthoracic echocardiography suggested a nail had possibly penetrated into the right ventricle. There was no associated pericardial effusion. In light of the potential for rapid deterioration, the patient was immediately transferred to the operating theatre where preparations for an exploratory midline sternotomy and thoracotomy were made.

An awake nasal fibreoptic tracheal intubation was performed using a 7.0mm internal diameter endotracheal tube. A 4mm bronchoscope and a 7-Fr Arndt™ wire-guided endobronchial blocker with a spherical cuff were prepared by coupling an Arndt Multiport Airway Adaptor to the bronchoscope and attaching them to the endotracheal tube. An endobronchial blocker with a spherical cuff was specifically utilised to ensure a proper fit should right lung or selective lobar separation be required. After induction of anaesthesia, the bronchoscope was advanced down the trachea until the carina was visualised and then advanced into the right main bronchus. The endobronchial blocker was advanced until the guide loop was seen to exit the end of the bronchoscope. The bronchoscope was retracted and the endobronchial blocker was placed in the right mainstem bronchus. Lung isolation was successfully achieved by inflating the balloon using the pilot balloon assembly kit.

**Figure 2:**
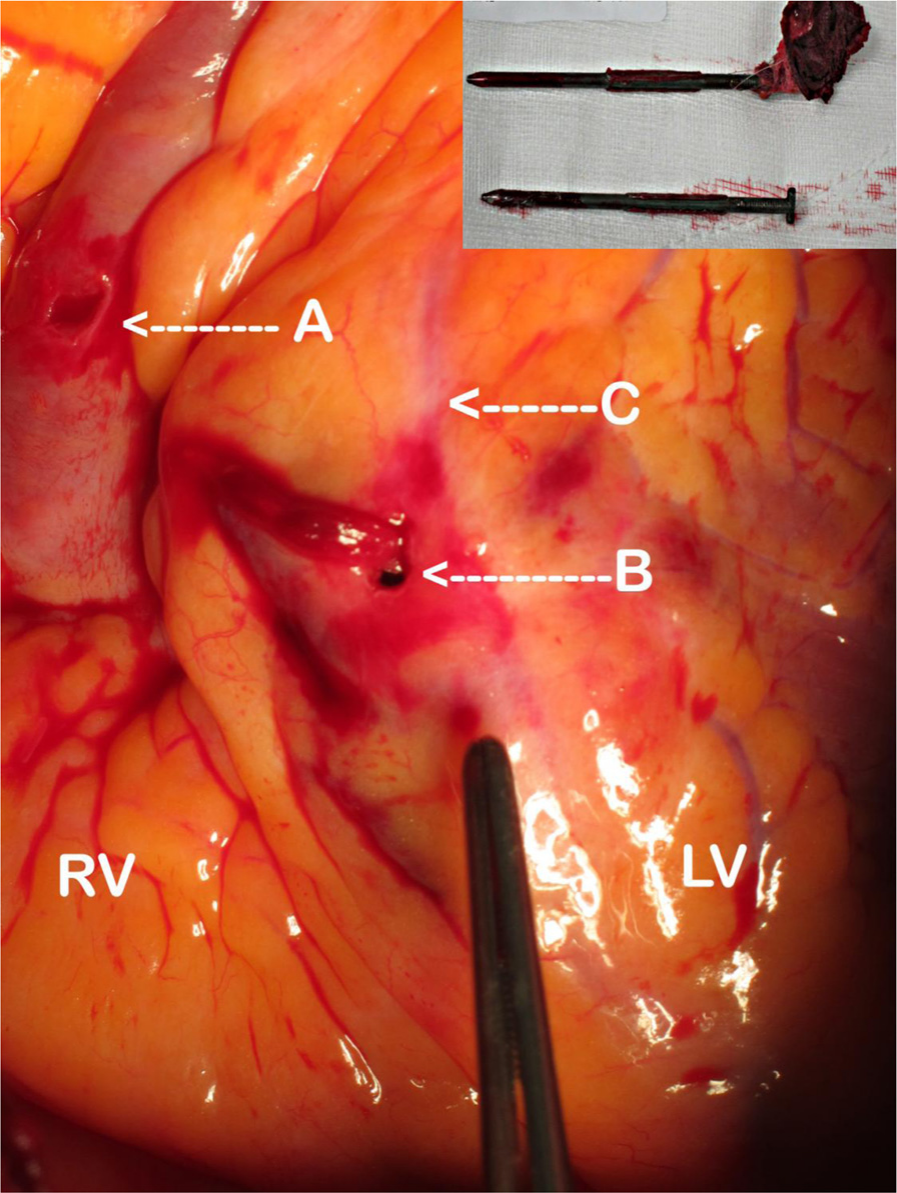
**Penetrating nails (insert) removed from the right ventricular free wall (A) and the right ventricle immediately at the junction with the right atrioventricular septum (B); (C) left anterior descending coronary artery.** RV, right ventricle; LV, left ventricle.

**Figure 3:**
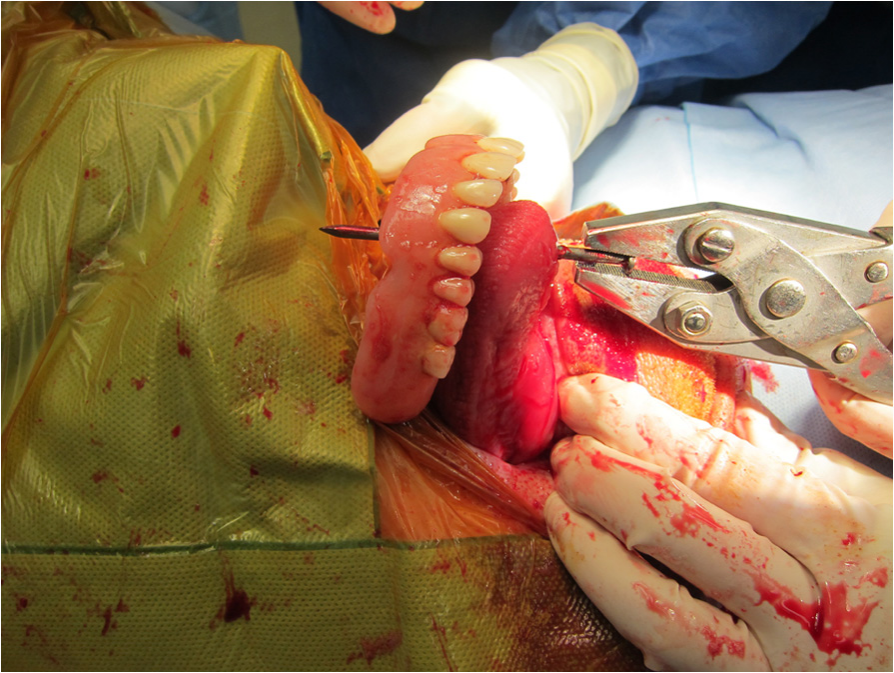
**Removal of the nail from the mouth using surgical pliers.** The nail was withdrawn through the hard palate, upper denture and tongue.

Surgery then commenced and a median sternotomy was made and two penetrating injuries were visualized on the right ventricular free wall and at the junction of the right atrioventricular septum. The left anterior descending artery was narrowly missed (Figure 2). A transoesophageal echocardiography probe, cautiously inserted through the side of the mouth, did not demonstrate any further cardiac defects. Cardiopulmonary bypass following systemic heparinization was initiated to facilitate surgical exposure. During cardiopulmonary bypass the endobronchial balloon was deflated, as lung isolation was not required, and cardiac repair was completed uneventfully. Removal of the nail from his mouth required a small incision into the hard palate and extraction using surgical pliers, removing the nail from his hard palate, upper denture and tongue (Figure 3). The patient made an excellent recovery and was discharged five days later to a psychiatric community hospital for ongoing psychological rehabilitation.

## Discussion

The airway considerations for accommodating the surgical requirement for lung separation and cardiopulmonary bypass in a patient with iatrogenically limited mouth opening are challenging. Our patient, who remained cooperative and haemodynamically stable, had a three-inch nail pinning his mouth shut, preventing conventional intubation via the orotracheal route. The potential for rapid haemodynamic deterioration due to the intrathoracic penetrating injury required urgent surgical exploration. The uncertainty in the location of the intrathoracic nails meant the exact nature of the surgical repair was not defined and provision for lung separation needed to be planned for. In this case, the chest X-ray failed to allow an adequate appreciation of the nail within the thorax. This occurred because the nail was imaged in short axis resulting in its appearance as a dot, which rendered it difficult to see. There was also a loss of the characteristic nail shape, which would have facilitated detection. A lateral chest X-ray would have been useful in this scenario but this, unfortunately, had not been ordered. Although transthoracic echocardiography suggested the nail had penetrated the right ventricle, the exact location of the nail was not visualized.

The decision between performing more advanced imaging such as computed tomography or proceeding straight to the theatre has been historically biased towards the latter by the threat of patient deterioration during the workup process. Multidetector computed tomography can generate high-resolution multiplanar and volumetric images that allow rapid localization of bleeding and assessment of intrathoracic structures and has been shown to improve the outcome of patients with penetrating cardiac injuries [1-5].

The standard approaches to achieve lung isolation for penetrating intrathoracic trauma are well described and include: selective single-lumen endobronchial tubes, double-lumen tubes (DLTs), and endotracheal tubes with bronchial blockers [6-9]. The requirement for heparin to enable the safe conduct of cardiopulmonary bypass necessitates selecting a minimally traumatic airway technique and one where bleeding can be controlled should it occur. The use of the Fogarty® embolectomy or bladder catheter as a bronchial blocker is mainly of historical interest and these devices have no role in lung separation strategies in the context of modern thoracic anaesthesia. The minimum safe tube length for endobronchial intubation via the nasal route is 40cm [4]. Single-lumen endobronchial tubes compatible with nasal intubation measuring 45 and 47cm in length are commercially available for both right- and left-sided endobronchial intubation (for example Rüsch™, Teleflex Medical), however double-lumen tubes cannot be inserted nasally, or used in patients with abnormal upper or lower airway anatomy, or fixed and/or limited mouth opening. A double-lumen tube was therefore not considered suitable for lung separation in this case.

The Univent™ bronchial blocker is a single-lumen endotracheal tube with an enclosed bronchial blocker. Whilst nasotracheal intubation and lung separation using a Univent™ tube has been previously reported [10], the large outer diameter makes nasal intubation difficult and traumatic, particularly in small patients, and its use in this setting is not advocated. As described in this case, when lung separation is critical and the orotracheal route is unsuccessful or not possible, we advocate nasotracheal intubation using a standard nasal endotracheal tube and deploying an independent bronchial blocker such as the wire-guided Arndt endobronchial blocker, Cohen™ endobronchial blocker or Fuji™ endobronchial blocker. The Arndt® blocker for nasotracheal intubation and lung isolation has been reported [11,12] but not in the context of penetrating chest trauma. A common problem for the thoracic anaesthetist is determining bronchoscope-endotracheal tube-blocker compatibility; therefore, a list of compatible endotracheal tubes, bronchoscopes and bronchial blockers is presented in Table 1.

**Table 1:** Bronchoscope tube compatibility chart

**Bronchoscope**	**2.8mm**	**3.3mm**	**4.0mm**	**4.9mm**	**5.9mm**
**SLT ID**	≥3.5	≥4.0	≥4.5	≥5.5	≥6.5
**SLT ID with 8, 9 Fr BB**	≥6.0	≥7.0	≥7.5	≥8.5	≥9.5
**SLT ID with 7 Fr BB**	≥5.5	≥6.0	≥6.5	≥7.5	≥8.5
**Univent™**	3.5	4.0	4.5	5.5	6.5
**Tracheostomy ID**	≥3.5	≥4.0	≥4.5	≥5.5	≥6.5
**Endobronchial tube ID**	≥3.5	≥4.0	≥4.5	≥5.5	≥6.5
**DLT (Fr)**	35, 37, 39, 41	35, 37, 39, 41	35, 37, 39, 41	41	NA

*SLT* single-lumen tube, *BB* bronchial blocker, *DLT* double-lumen tube, *ID* internal diameter, *NA* not applicable, *Fr* French. Single-lumen endobronchial tubes are considered identical to SLTs.

**Figure 4:**
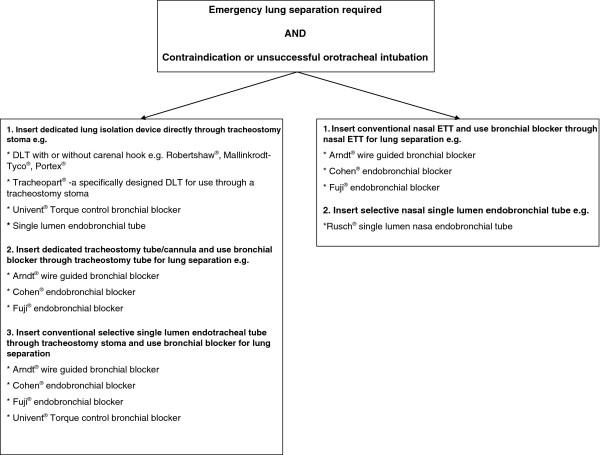
**An algorithm for emergency lung separation when orotracheal airway management is not possible.** DLT, double-lumen tube; ETT, endotracheal tube.

Importantly, when there is a need for rapid lung isolation where conventional orotracheal or nasal intubation is not possible, a surgical tracheostomy can be performed under direct vision, and with minimal haemodynamic disturbance under local anaesthetic. Double-lumen tubes and endobronchial blockers can be inserted via the tracheostomy to achieve lung separation [13-16], in addition to commercially available lung isolation tracheostomy tubes (Tracheopart™). An algorithm for emergency lung separation when orotracheal airway instrumentation is not possible is presented in Figure 4.

## Conclusions

In conclusion, we report a rare case of deliberate self-harm in which three three-inch nails were fired from a nail gun resulting in mandibular fixation and right cardiac ventricular penetration. The patient required emergency cardiopulmonary bypass to repair penetrating injuries to the right ventricle followed by the removal of the nail from the floor of the mouth. This rare case offers airway strategies to accommodate the surgical requirement for lung separation for penetrating chest trauma in a patient with iatrogenically limited mouth opening. The principles of successful lung separation in patients where the orotracheal route is not possible require a detailed understanding of the tracheobronchial anatomy, familiarity with the fiberoptic bronchoscope, and expertise with both DLTs and bronchial blockers.

## Consent

Written informed consent was obtained from the patient for publication of this case report and any accompanying images. A copy of the written consent is available for review by the Editor-in-Chief of this journal.

## Competing interests

The authors declare that they have no competing interests.

## Authors’ contributions

LW was the principle cardiac anaesthetist involved in management of the case. In addition, he was responsible for the review of all literature, photographing clinical pictures, obtaining patient consent, and the writing of the manuscript. HL was the provisional fellow involved in management of the patient and was responsible for the literature review, medical data collection, and the writing of the manuscript. COT was the anaesthetist responsible for collation of all transoesphageal images, anaesthesia data collection, and the writing of the manuscript. ST was responsible for assisting with the writing of the manuscript**.** CK was the anaesthetist responsible for assisting with airway management and the writing of the manuscript. PP was the medical perfusionist involved in management of the case. He assisted with collation of all transoesphageal images, anaesthesia data collection, and the writing of the manuscript. All authors read and approved the final manuscript.
